# The anabolic action of intermittent parathyroid hormone on cortical bone depends partly on its ability to induce nitric oxide‐mediated vasorelaxation in BALB/c mice

**DOI:** 10.1002/cbf.3164

**Published:** 2016-02-01

**Authors:** S Gohin, A Carriero, C Chenu, AA Pitsillides, TR Arnett, M Marenzana

**Affiliations:** ^1^ Department of BioengineeringImperial College LondonLondonUK; ^2^ Department of Biomedical EngineeringFlorida Institute of TechnologyFloridaUSA; ^3^ Department of Cell and Developmental BiologyUniversity College LondonLondonUK; ^4^ Department of Comparative Biomedical SciencesRoyal Veterinary CollegeLondonUK; ^5^ Kennedy Institute of RheumatologyUniversity of OxfordOxfordUK

**Keywords:** parathyroid hormone, bone blood flow, nitric oxide, vasodilation, cortical bone

## Abstract

There is strong evidence that vasodilatory nitric oxide (NO) donors have anabolic effects on bone in humans. Parathyroid hormone (PTH), the only osteoanabolic drug currently approved, is also a vasodilator. We investigated whether the NO synthase inhibitor L‐NAME might alter the effect of PTH on bone by blocking its vasodilatory effect. BALB/c mice received 28 daily injections of PTH[1–34] (80 µg/kg/day) or L‐NAME (30 mg/kg/day), alone or in combination. Hindlimb blood perfusion was measured by laser Doppler imaging. Bone architecture, turnover and mechanical properties in the femur were analysed respectively by micro‐CT, histomorphometry and three‐point bending. PTH increased hindlimb blood flow by >30% within 10 min of injection (*P* < 0.001). Co‐treatment with L‐NAME blocked the action of PTH on blood flow, whereas L‐NAME alone had no effect. PTH treatment increased femoral cortical bone volume and formation rate by 20% and 110%, respectively (*P* < 0.001). PTH had no effect on trabecular bone volume in the femoral metaphysis although trabecular thickness and number were increased and decreased by 25%, respectively. Co‐treatment with L‐NAME restricted the PTH‐stimulated increase in cortical bone formation but had no clear‐cut effects in trabecular bone. Co‐treatment with L‐NAME did not affect the mechanical strength in femurs induced by iPTH. These results suggest that NO‐mediated vasorelaxation plays partly a role in the anabolic action of PTH on cortical bone. © 2016 The Authors. Cell Biochemistry and Function published by John Wiley & Sons, Ltd.

## Introduction

The cardiovascular action of parathyroid hormone (PTH) was first observed in 1925, soon after its discovery as a hypercalcaemic agent 1. Decades later, PTH was shown to decrease arterial blood pressure and increase cardiac output, with marked increases in coeliac, coronary and renal blood flows and corresponding decreases in vascular resistance 2, 3. PTH also has an opposing hypertensive action, secondary to its potent hypercalcemic effect 4.

Since the discovery of the powerful osteoanabolic effect of intermittently administrated PTH (iPTH)^5^, research has focused mainly on its skeletal effects; iPTH remains the most potent bone anabolic drug available at present 6. There is good evidence that a key mechanism underlying the osteogenic action of PTH is the suppression of sclerostin production 7, an inhibitor of Wnt signalling that is predominantly produced by osteocytes and hypertrophic chondrocytes 8. Further evidence that the effect of PTH on bone is mediated by bone cells is provided from genetic studies where selective activation or disruption of PTH receptor type 1 (PTHR1) signalling in osteocytes modified the anabolic and catabolic responses induced by PTH in mice 9, 10. One key aspect underpinning the complex *in vivo* role of PTH, which merits consideration when exploring its mode of action, is its pivotal dependence on mode of administration: although intermittent injections of PTH increase bone mass, continuous infusion stimulates osteoclastic resorption and decreases bone mass^11^, ^12^. Surprisingly, the direct effect of PTH on primary rodent osteoblasts *in vitro* is to strongly inhibit osteogenic differentiation and bone formation 13, 14. This observation suggests that an additional indirect action of the hormone could contribute to the osteoanabolic effect of PTH on bone *in vivo*.

In recent years, considerable evidence has emerged in support of the critical role of the vascular supply in the control of bone formation and turnover 15, 16, 17, 18, 19. The possibility that some osteogenic effects of iPTH rely on its vascular function has yet, however, to be fully explored. PTHR1 is expressed by many cell types, including endothelial and vascular smooth muscle cells 20, 21. PTH has recently been shown to increase blood perfusion of bone marrow in the mouse tibia in a manner that correlated strongly with bone remodelling parameters 22. The same study also showed that iPTH moderately increased intraosseous angiogenesis and blood vessel remodelling within the bone marrow. Based on these results and those of a previous study in rats 23, the authors concluded that the combination of vascular remodelling and vasotonic action underpins the increase in bone perfusion induced by iPTH. Both the vascular remodelling and the osteoanabolic effects of iPTH have been shown to depend on the potent angiogenic factor, vascular endothelial growth factor (VEGF), whose production is directly stimulated by PTH in bone 23, and bone and endothelial cells 24, 25. A recent report showed that VEGF signalling was involved in mediating PTH‐induced vasorelaxation in isolated femoral principal nutrient arteries (PNAs), suggesting that it may also partly mediate the osteoanabolic effect of PTH *in vivo* 26. Interestingly, this study also revealed a strong dependency of PTH vasodilatory effect in PNAs on nitric oxide synthase (NOS) activity 26. Although these studies suggest that the osteoanabolic action of iPTH may depend on its vascular effects, it is still unknown whether the acute vasodilatory effect of iPTH could be involved in augmenting bone blood flow during the course of treatment. Similarly, whilst the osteoanabolic effects of several NO donor drugs are well documented 27, it is not known whether the transient vasorelaxation induced daily by these drugs constitutes an essential component of their mechanism of action. The physiological need for increasing blood supply in response to the metabolic demands of increased bone formation and turnover is well‐documented 16. In the present study we investigated whether the acute vasodilatory effect of PTH contributes to limb blood flow, bone perfusion and, ultimately, to its osteoanabolic action.

## Material and Methods

### Drugs and reagents

Human PTH (1–34), bovine serum albumin (BSA), N_ω_‐nitro‐L‐arginine methyl ester hydrochloride (L‐NAME), procion red MX‐5B, calcein and xylenol orange were purchased from Sigma (UK). Phosphate buffered saline solution (PBS) was purchased from VWR (UK). All drugs were used at a dose required to produce the desired pharmacological effects in mice, according to previous protocols 28, 29.

### Experimental design

Male BALB/c mice, aged 10–12 weeks, were purchased from Charles River Laboratories, Ins (UK) and maintained under a controlled temperature (21 °C) and lighting with 12 h/12 h light/dark cycle. They received a standard mouse diet and water *ad libitum*. Mice were randomized into four groups (8 mice/group): (1) a control group receiving daily intraperitoneally (i.p.) injections of PBS; (2) a group injected daily i.p. with human PTH (1–34) alone (80 µg/kg/day, dissolved in 1 mM HCl containing 0.2% bovine serum albumin); (3) a group injected daily subcutaneously (s.c.) with L‐NAME alone (30 mg/kg/day); and (4) a group co‐administered daily with PTH and L‐NAME at the doses specified above. All groups were treated for 28 days. The body weight of all mice was measured every day.

To measure bone formation rates, mice were injected s.c. with calcein (15 mg/kg), xylenol orange (90 mg/kg) and calcein again, respectively before the beginning of treatments, 7 days and 2 days before the end of the study.

Blood perfusion in one hind limb was monitored during the administration of treatments (acute effect) by laser Doppler imaging (LDI) under general anaesthesia. All mice underwent one LDI measurement on the first day of dosing and then each mouse was imaged on at least two further occasions, throughout the duration of the study. The acute effect of PTH on intraosseous cortical bone perfusion was assessed by intravenous infusion of a bolus of a fluorescent tracer in two additional groups of naïve mice (*n* = 4), injected either with human PTH (1–34) (80 µg/kg) or PBS.

At the end of the study, mice were sacrificed by neck dislocation, left femurs were dissected, fixed in 10% buffered formalin for 48 h and stored in 70% ethanol for micro‐computed tomography (microCT) imaging and undecalcified histomorphometry. Right femurs were dissected and immediately frozen for subsequent mechanical tests.

All procedures were performed in accordance with the principles and guidelines established by the Animals Scientific Procedures Act and UK Home Office regulations (PPL: 70/7444).

### Measurements of whole hind limb and intraosseous cortical bone perfusion

Two days before hind limb perfusion measurements, the inner side of the hind limb to be imaged was shaved with a depilatory cream under general anaesthesia. At the time of the measurement, mice were anesthetized with 2% isoflurane. Body temperature was maintained between at 36 and 37 °C with a feedback‐controlled heating pad (507220F, Harvard Apparatus, UK). Hind limb perfusion was measured using a laser Doppler imaging (LDI) flow meter (MoorLDI2 Imager, Moor Instruments Ltd, UK). The instrument measures the distribution of blood flux (combination of velocity and concentration of red blood cells) in arbitrary units for a selected region of interest (19 mm × 27 mm) with a special resolution of 100 µm/pixel. The estimated maximum depth of detectable vessels (in soft connective tissue) was estimated to be 2–3 mm (laser wavelength = 830 nm).

For the perfusion measurements, reagents were administered in the same order as during the chronic study: first PBS or L‐NAME was injected subcutaneously and then PBS or PTH (1–34) was injected i.p. within 10 min after the first injection. Perfusion was recorded continuously for 30 min (starting 10 min before the first injection).

Vascular tracer uptake into the lacuno‐canalicular porosity, an established method to measure intraosseous perfusion 30, 31, was assessed by injecting a bolus of procion red solution (0.8% in PBS), equal to 10% of the body weight, into the tail vein of naïve mice. The procion red bolus was given to two groups of restrained, conscious mice (*n* = 4) immediately after i.p. injection of either PTH (1–34) or PBS. Mice were allowed to roam freely in their cages for 5 min after the injection, and then sacrificed by neck dislocation. Femurs were immediately dissected and fixed in 70% ethanol. Procion red uptake in cortical bone was examined in non‐decalcified histological sections of the femoral mid‐diaphysis using the protocol described below.

### Assessment of microstructure and volumetric bone mineral density by microCT

The microstructure and volumetric bone mineral density (vBMD) of femurs were assessed by high‐resolution microCT (5 µm/pixel, 50 kV, 200 μA, Skyscan 1172, Belgium). Image reconstruction was performed by NRecon software (Skyscan) and bone parameters were analysed in 2D and 3D using CT.An software (Skyscan). The cortical and trabecular compartment within the femoral metaphysis and the vertebral body were determined by manual contouring. Specifically, the trabecular bone within the secondary spongiosa was analysed in the femoral metaphysis, a volume extending 1 mm proximally from the primary spongiosa limit (200 tomograms). In the vertebral body, the entire trabecular content extending between the two cortical plates was contoured and analysed. The threshold values for the microCT analysis of trabecular bone were chosen between 60 and 255, while they were between 100 and 255 for cortical bone. The following standard histomorphometric parameters were evaluated in trabecular bone using a 3D‐based analysis software (Ct.An, Skyscan): bone volume fraction (BV/TV, computed as the ratio of bone volume, BV, over tissue volume, TV), trabecular thickness (Tb.Th), trabecular space (Tb.Sp), trabecular number (Tb.N) and 3D structure model index (SMI), trabecular pattern factor (Tb.Pf), trabecular connectivity density (Conn.D).

Cortical bone in the mid‐diaphysis was evaluated in 50 tomograms located at 50% of the total length of femur. The following parameters were evaluated in cortical bone using Ct.An and Matlab software: tissue volume (TV), bone volume (BV), average cross‐sectional tissue area (Tt.Ar), average cross‐sectional bone area (Ct.Ar), average cross‐sectional cortical thickness (Ct.Th), maximum moment of inertia (Imax), minimum moment of inertia (Imin), polar moment of inertia (Ipol), volumetric bone mineral density (vBMD) and percentage porosity.

### Non‐decalcified bone histomorphometry

After microCT scanning, femurs were dehydrated and embedded in methyl methacrylate using an established cold embedding protocol which preserves tartrate resistant acid phosphatase (TRAP) enzymatic activity 32. Thicker cross sections of the femur mid‐diaphysis (70–100 µm) were obtained using a slow‐speed rotating saw (Isomet, Buehler, USA) while thinner coronal sections of the femur distal metaphysis (8 µm thick) were cut with a microtome (RM2255, Leica, Germany). To determine the osteoclast surface per bone surface (Oc.S/BS), sections were stained for TRAP (Leucognost SP kit, Merck) and counterstained with Weigert's haematoxylin. To determine the osteoid surface per bone surface (Ost.S/BS), sections were stained using a modified Goldner's trichrome protocol.

Fluorescent labels were visualized using a confocal laser scanning microscope (TCS SP5, Leica, Germany) equipped with 488 nm (for calcein) and 561 nm (for xynelol orange) lasers. For cortical bone, double calcein‐labels were measured using an Osteometrics image analysis software system, and the following parameters were evaluated: mineralizing surfaces (MS/BS, %), computed as the last calcein labelled surfaces (MS, µm^2^) normalized to all bone surfaces (BS, µm^2^); mineral apposition rate (MAR, µm/day), measured as average distance between adjacent pairs of calcein labels divided by the number of days elapsed between the administration of both labels (26 days); bone formation rates (BFR/BS, µm^3^/µm^2^/d), derived by multiplying MS/BS by MAR. For trabecular bone, only mineral apposition rate was evaluated using a dedicated open source plugin for Image J developed in our laboratory. We measured the distance between the xynelol orange label and the last calcein label (5 days between both labels). All histomorphometric parameters were quantified using a semi‐automated programme, developed by Dr. van't Hof 33.

Procion red uptake in the mid‐shaft cortical bone was assessed in the thick sections (70–100 µm) by confocal microscopy (excitation laser = 561 nm, emission filter = 610 nm). Briefly, a region of interest (ROI) was manually drawn within the postero‐medial quadrant of the mid‐diaphysis cortical bone. The average fluorescence intensity of each ROI was normalized by the area of the ROI and was used a measure of the average concentration of the tracer per unit area of cortical bone.

### Assessment of bone mechanical properties

For biomechanical analysis, right femurs were stored in gauze soaked in saline solution at −80 °C and thawed before analysis at room temperature. They were tested until fracture by three‐point bending using an Instron testing machine (5866, Norwood, MA, USA). Femurs were loaded at the mid‐diaphysis in their antero‐posterior direction with a deflection rate of 1 µm/s while load was measured using a calibrated 50‐N load cell. The loading span (i.e. the distance between the two rods supporting the femur shaft) was fixed to 9 mm. Femora were kept moist at all times. Force–deflection curves were analysed with a custom programme in Matlab (MathWorks Inc, MA, USA). Geometry parameters, including the mid femur cross‐section dimensions were measured from the microCT images of the femur mid‐diaphysis.

Bending stress (σ) was computed from the force according to the equation: σ = FLd/4I_min_, where F is the force, L is the loading span, d is the antero‐posterior diameter and I_min_ is the second moment of inertia from the lateromedial axis (πd^4⁄64); bending strain (e) was calculated according to the equation: e = deflection × 6 × d / (L^2); 34. The following set of parameters—regarded as extrinsic mechanical properties—was directly measured from the force–deflection curves: bending stiffness (S, slope of the linear elastic deformation), yield load (F_yield_, force limit between the elastic and plastic deformation), ultimate load (F_ult_, maximum force sustained) and breaking load (F_break_, force at which the bone breaks). An additional set of parameters—regarded as intrinsic mechanical properties—was computed using the standard beam theory from the stress–strain curves derived from the force–deflection curves 34. These included: bone elastic modulus (E, slope of the linear elastic deformation in the stress‐strain curve), yield stress (σ_yield_, stress limit between the elastic and plastic deformation), ultimate stress (σ_ult_, maximum stress sustained) and breaking stress (σ_break_, stress at which the bone breaks). Finally, total and plastic (yield) work to fracture, as well as their ratio, were calculated as a measure of bone toughness.

### Statistical analysis

Data are reported as mean ± standard deviation (SD). Results were analysed by one‐way ANOVA, followed by Dunnett's post hoc test. Tukey's post hoc test was used to compare all possible pairings in the four groups. The homoscedasticity requirement (equal variances across groups) for the applicability of one‐way ANOVA was established by the Bartlett's and the Brown–Forsythe test (which was not significant for all sets of parameters tested). Statistical analysis was computed using Prism v.6 software (GraphPad, USA). A two‐tailed *P* value less than 0.05 was regarded as statistically significant.

## Results

### iPTH induces acute but not chronic increases in both intra‐cortical and hind limb perfusion that are abolished by L‐NAME co‐administration

**Figure 1:**
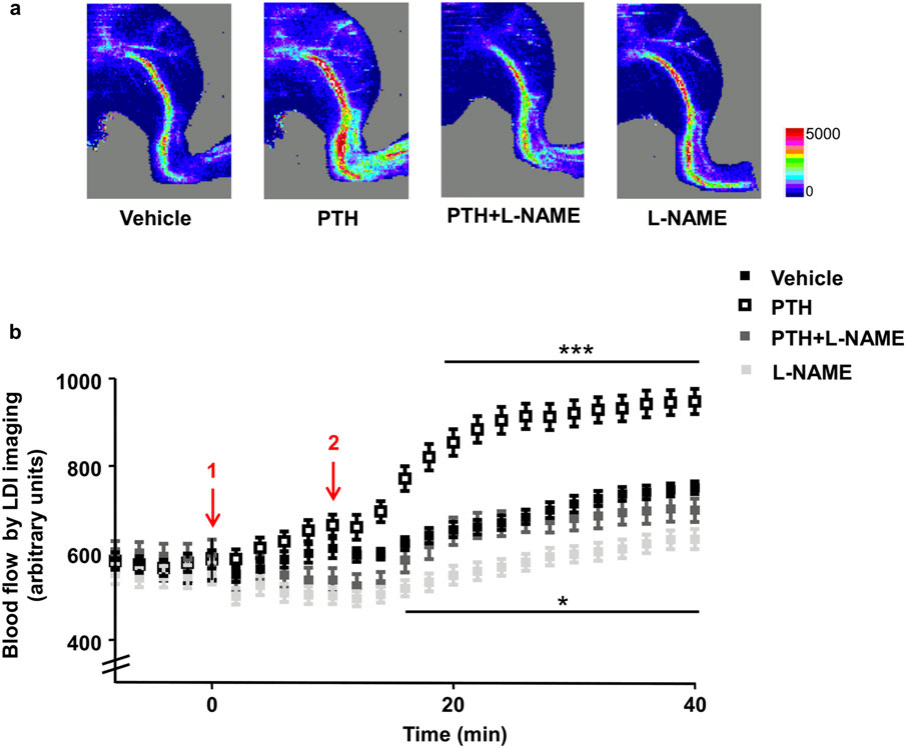
Intermittent PTH increases bone blood flow acutely in BALB/c mice. (a) Representative images of the mouse hind limb perfusion, obtained by laser Doppler imaging (LDI). Images acquired 10 min after administration of vehicle, PTH 1–34 (80μg/kg), L‐NAME (30 mg/kg) or the combination PTH + L‐NAME. The colours in the heat maps indicate the maximum (red) and the minimum (blue) level of perfusion expressed in arbitrary units representing the total blood flux (combination of velocity and concentration of red blood cells). (b) Time course graph of the average hind limb perfusion monitored by LDI for PTH and L‐NAME, alone or in combination. Arrows: 1—Vehicle or L‐NAME injection; 2—vehicle or PTH. Values are presented as means ± standard errors of the mean (S.E.M.); *n* = 10–14 mice/group; *P < 0.05 versus Control; *** *P* < 0.001 versus Control

The mean body weight of mice did not change during the 4‐week treatment, and was similar among the four treatment groups (vehicle = 25.3 ± 0.4g, iPTH = 25.5 ± 0.5g, iPTH + L‐NAME = 25.4 ± 0.3g, L‐NAME = 25 ± 0.8g, mean ± SD, *n* = 10–14). Acute changes in hind limb perfusion were measurable by LDI on the first day of dosing, with PTH producing significant elevation (over 30%; *P* < 0.001) in perfusion within 10–15 min compared to the vehicle‐treated controls, which was sustained for ≥30 min (Figure 1 a, b). Notably, this acute response was reproduced in randomly selected mice after any of the daily doses of PTH throughout the 4‐week period (approximately two random measurements in each mouse, besides the initial one). While L‐NAME alone had little decreases compared to the control, its co‐administration completely blocked the PTH‐related increases in vasorelaxation (Figure 1 a, b). However, at the end of the 4‐week treatment, no measurable chronic changes in hind limb blood perfusion were evident 24 h after the last dosing in any groups compared to vehicle controls (vehicle = 641 ± 112, PTH = 775 ± 215, iPTH + L‐NAME = 734 ± 117, L‐NAME = 678 ± 122, perfusion expressed in arbitrary units, mean ± SD, *n* = 10–14). These data indicate that iPTH induces acute, but not chronic increases in hind limb perfusion which are abolished by co‐administration of L‐NAME, suggesting that they are mediated by increases in NO production.

**Figure 2:**
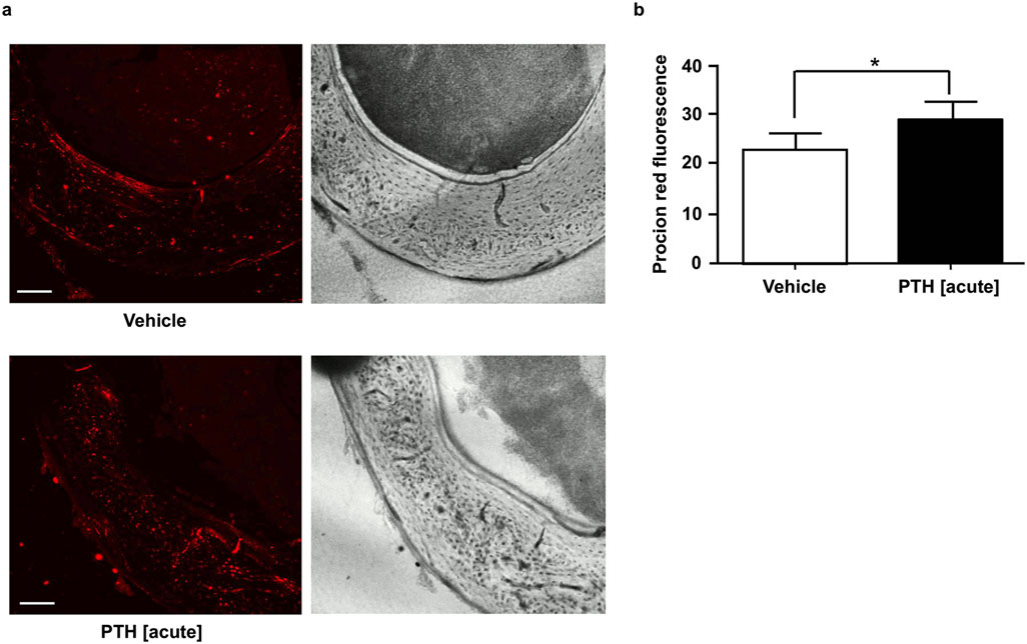
Acute effect of PTH treatment on lacunar–canalicular perfusion. (a) Representative confocal micrographs of non‐decalcified sections of the femoral mid‐diaphysis showing the uptake of fluorescent tracer in cortical bone 5 min after injection of vehicle (control) and PTH 1–34 (80 µg/kg). Scale bar = 100 µm. (b) Average tracer fluorescence within a region of interest manually drawn within the postero‐medial quadrant of the mid‐diaphysis cortical bone. Values are mean ± SD, *n* = 4 mice/group; * *P* < 0.05 versus Control

This iPTH‐induced hind limb perfusion might not necessarily be translated to changes in intra‐cortical bone perfusion; therefore, in order to relate whole hind limb perfusion (measured by LDI) to direct intracortical bone perfusion, we used confocal microscopy to assess uptake of procion red in cortical bone of the femoral mid‐diaphysis. Our data revealed that PTH significantly increases procion red average fluorescence (proportional to its concentration) in femoral cortical bone within 5 min of injection (Figure 2 a, b). This suggests that PTH injection induces both acute elevation in blood perfusion and a closely linked increase in tracer perfusion within the femoral cortical bone tissue.

### iPTH induced increases in cortical bone volume and bone formation rate that are partially blunted by L‐NAME co‐administration

**Figure 3:**
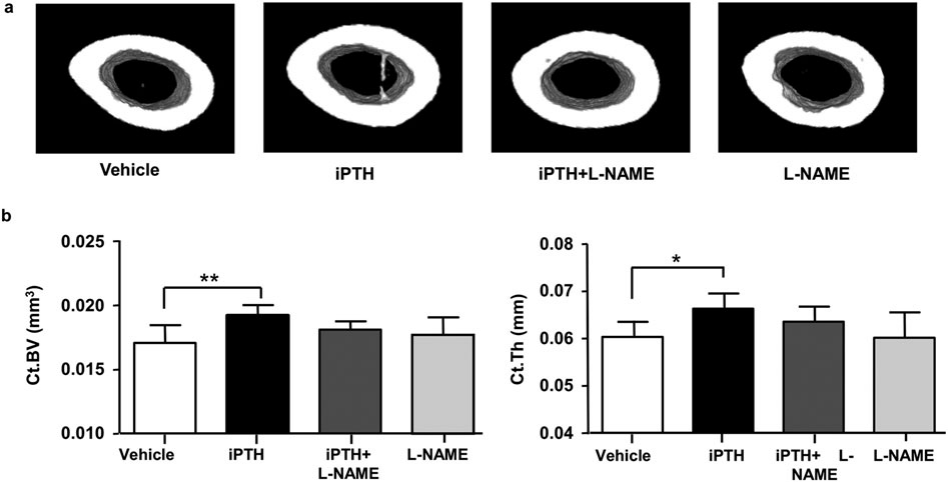
L‐NAME decreases the anabolic action of intermittent PTH on cortical bone volume. (a) Three‐dimensional (3D) renderings of 2‐mm segments of the femur mid‐diaphysis imaged by microCT at the end of 28‐day treatment with vehicle (control), iPTH and L‐NAME, alone or in combination. (b) Structural parameters computed from the microCT scans: mid‐diaphysis cortical bone volume (Ct.BV) and cortical bone thickness (Ct.Th). Values are mean ± SD, *n* = 7–8 mice/group; * *P* < 0.05 versus Control; ** *P* < 0.01 versus Control

Cortical bone geometry and bone quality parameters in the femoral mid‐diaphysis were measured by microCT. As expected, significantly increased cortical bone volume (+12%) and average thickness (+10%) were observed in mice treated with iPTH compared to vehicle‐treated mice (Figure 3 a, b). Moreover, cortical tissue volume was slightly higher in iPTH‐treated mice compared with vehicle treated mice, although this was not significant (*P* = 0.059). These iPTH‐induced changes were abrogated by co‐administered L‐NAME treatment, which effectively restrained cortical bone geometry to levels evident in control mice. L‐NAME treatment alone had no effect (Figure 3 a, b). Additional measures of mid‐shaft geometry (Table 1) were also significantly elevated by iPTH compared with control, including Imin (+18%) and Ipol (+16%), and these were similarly reduced to basal control levels by co‐administration of L‐NAME. MicroCT analysis of bone quality indexes, including bone mineral density (BMD) and cortical porosity, showed that these were unchanged in the experimental groups (Table 1).

**Table 1:** MicroCT analysis of femoral mid‐diaphysis cortical bone and distal metaphysis trabecular bone. Femora were dissected at the end of the 28‐day dosing and analysed by micro‐CT. Bone parameters are shown for the four treatment groups (*n* = 7–8 mice/group): Vehicle (control), iPTH, iPTH + L‐NAME and L‐NAME alone

**Cortical bone parameters**	**Control**	**iPTH**	**iPTH + L‐NAME**	**L‐NAME**
Tissue volume, TV (mm^3^)	0.028 ± 0.001	0.030 ± 0.001	0.028 ± 0.001	0.029 ± 0.001
Cross‐sectional tissue area, Tt.Ar (mm^2^)	0.105 ± 0.009	0.112 ± 0.004	0.107 ± 0.004	0.107 ± 0.007
Cross‐sectional bone area, Ct.Ar (mm^2^)	0.064 ± 0.005	0.072 ± 0.003^a^	0.068 ± 0.002	0.066 ± 0.005
Imax (mm^4^)	0.23 ± 0.04	0.27 ± 0.03	0.24 ± 0.02	0.24 ± 0.03
Imin (mm^4^)	0.10 ± 0.02	0.12 ± 0.01^a^	0.11 ± 0.01	0.11 ± 0.01
Ipol (mm^4^)	0.0016 ± 0.0003	0.0019 ± 0.0001^a^	0.0017 ± 0.0001	0.0017 ± 0.0002
vBMD (g/cm^3^)	1.269 ± 0.017	1.278 ± 0.010	1.276 ± 0.013	1.279 ± 0.010
Cortical porosity (%)	0.04 ± 0.02	0.04 ± 0.05	0.02 ± 0.03	0.05 ± 0.05
**Trabecular bone parameters**	**Control**	**iPTH**	**iPTH + L‐NAME**	**L‐NAME**
Trabecular space, Tb.Sp (mm)	0.18 ± 0.01^b^	0.21 ± 0.01^a^, ^b^, ^c^	0.23 ± 0.01^a^, ^c^	0.19 ± 0.01^b^
Trabecular pattern factor, Tb.Pf (mm^−1^)	20.9 ± 2.5^b^	21.3 ± 1.8^b^	24.5 ± 2.2^a^	22.7 ± 1.9
Connectivity density, Conn.D (mm^‐3^)	290.4 ± 35.5^b^	183.4 ± 25.0^a^, ^b^, ^c^	144.8 ± 18.6^a^, ^c^	273.7 ± 37.7^b^

Values are mean ± SD;

a *P* < 0.05 versus control;

b *P* < 0.05 versus iPTH + L‐NAME;

c *P* < 0.05 versus L‐NAME.

**Figure 4:**
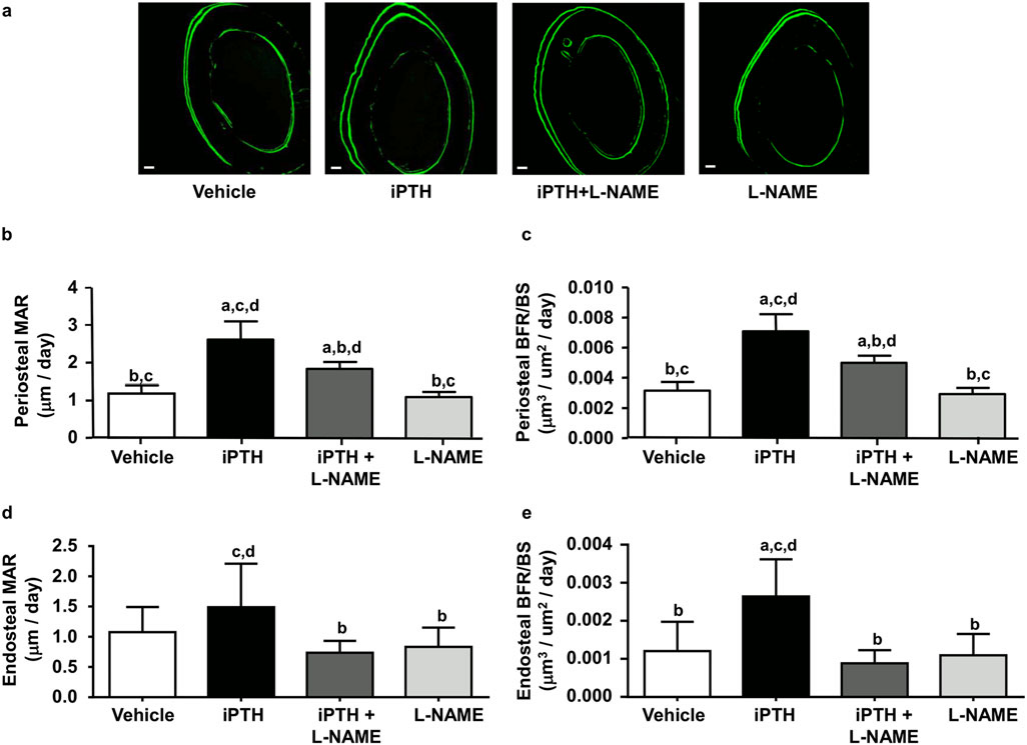
L‐NAME reduces iPTH‐induced increases in cortical bone formation rate. (a) Representative confocal micrographs of non‐decalcified sections of the femoral mid‐diaphysis, showing the calcein double labels (26 days between both labels) marking bone apposition during the 26 days of dosing with vehicle (control), iPTH and L‐NAME, alone or in combination. Scale bar = 50 µm. (b) Bar graphs of the average periosteal mineral apposition rate (MAR) and (c) periosteal bone formation rate (BFR/BS) for the four groups. (d) Bar graphs of the average endosteal mineral apposition rate (MAR) and (e) endosteal bone formation rate (BFR/BS) for the four groups. Values are mean ± SD, *n* = 7–8 mice/group; ^a^ *P* < 0.05 versus control; ^b^ *P* < 0.05 versus iPTH; ^c^ *P* < 0.05 versus iPTH + L‐NAME, ^d^ *P* < 0.05 versus L‐NAME

Because of the differences in blood supply between the bone periosteal surfaces (normally well vascularized) and the endosteal surfaces (poorly supplied) 35, we investigated whether the modulation of vascular function induced by the different treatments might produce differing levels of osteoblastic activation in each of these two envelops. Quantifying bone formation rate in the whole bone and separately in periosteal and endosteal bone compartments revealed that the whole bone response more closely matched the periosteal response (Figure 4 a–c). Intermittent PTH treatment significantly increased mineral apposition rate (MAR) and bone formation rate (BFR/BS) on the periosteal surfaces compared to vehicle‐treated controls (+92% and +130%, respectively) (Figure 4 a–c). These iPTH‐induced increases in MAR and BFR/BS were significantly suppressed by co‐administration of L‐NAME (by 40%). These levels were nevertheless still significantly elevated when compared to either vehicle (+39%) or L‐NAME‐treated groups (+48%), despite the lack of any modification in MAR or BFR/BS by L‐NAME treatment alone (Figure 4 a–c). Conversely, BFR/BS was elevated on the endosteal surfaces in the iPTH (+120%) and L‐NAME alone (+142%) groups compared to control, but not in the iPTH + L‐NAME group (Figure 4e), where it was significantly reduced in comparison with the iPTH group (−66%; Figure 4e).

In summary, L‐NAME co‐administration either partially or completed inhibited the increase in the rate of bone formation induced by iPTH, and this inhibition was dependent upon the cortical bone envelope (periosteal or endosteal) of the femur midshaft.

### Co‐administration of L‐NAME with PTH reduces trabecular bone volume

**Figure 5:**
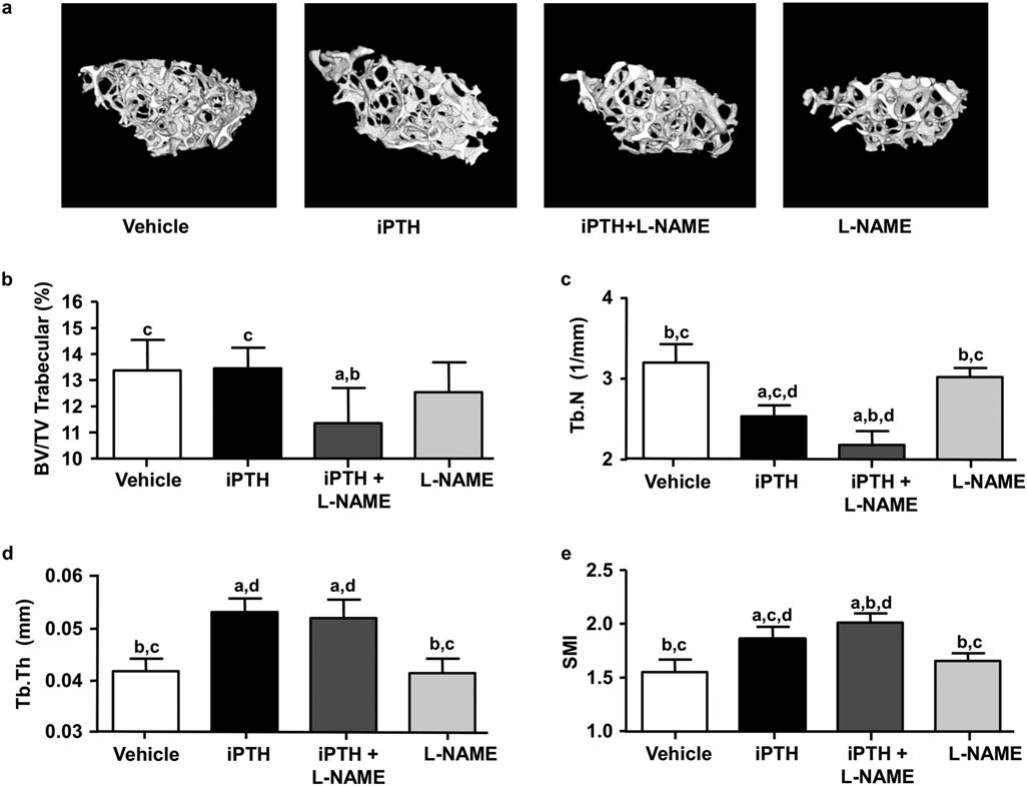
Effects of PTH and L‐NAME on trabecular bone architecture in femurs. (a) Three‐dimensional (3D) rendering of microCT scans of trabecular bone in secondary spongiosa of the distal femur in mice treated with vehicle (control), iPTH and L‐NAME, alone or in combination for 28 days. The bar graphs show some of the parameters obtained from the 3D analysis of the trabecular architecture, including (b) the bone volume fraction (BV/TV), (c) the number of trabeculae (Tb.N), (d) the trabecular thickness (Tb.Th) and (e) the structure model index (SMI). Values are mean ± SD, *n* = 7–8 mice/group; ^a^ *P* < 0.05 versus control; ^b^ *P* < 0.05 versus iPTH; ^c^ *P* < 0.05 versus iPTH+L‐NAME, ^d^ *P* < 0.05 versus L‐NAME

We then analysed trabecular bone in the femoral metaphysis in order to determine whether similar sensitivity to L‐NAME and iPTH was evident in this highly vascularized compartment. In contrast to the anabolic effect of PTH in cortical bone, microCT analysis of the femoral metaphysis showed that iPTH treatment in BALB/c mice induced a decrease in trabecular number (Tb.N; −21%) and augmented trabecular thickness (+27%) without any modification in total trabecular bone volume fraction (BV/TV; Figure 5 a–d). L‐NAME treatment alone did not significantly affect any trabecular bone parameter, compared to controls (Figure 5 a–e) but its co‐administration with iPTH resulted in decreased BV/TV (−15%), greater reduction in Tb.N (−32%) and further enhanced Tb.Th (+24%) compared to vehicle‐treated controls (Figure 5 a–d). iPTH also elevated the structural model index (SMI; +20%), which was further increased by L‐NAME co‐administration (Figure 5 e). Additional measures of trabecular bone parameters (Table 1) were also significantly modified by iPTH and by L‐NAME. Tb.Sp was increased by iPTH compared to control (+16%) and further increased by L‐NAME co‐administration. iPTH co‐administrated with L‐NAME increased Tb.Pf compared to controls (+17%) and iPTH alone (+15%). Conn.Dn was reduced by PTH treatment compared to control (−37%), and greater reductions were observed with L‐NAME co‐administration (−50%; Table 1). Similar microstructural changes were observed in the trabecular bone of L5 vertebrae (data not shown), indicating a compartment‐specific (trabecular versus cortical bone) but not a site‐specific (appendicular versus axial skeleton) sensitivity to iPTH and L‐NAME treatment.

**Figure 6:**
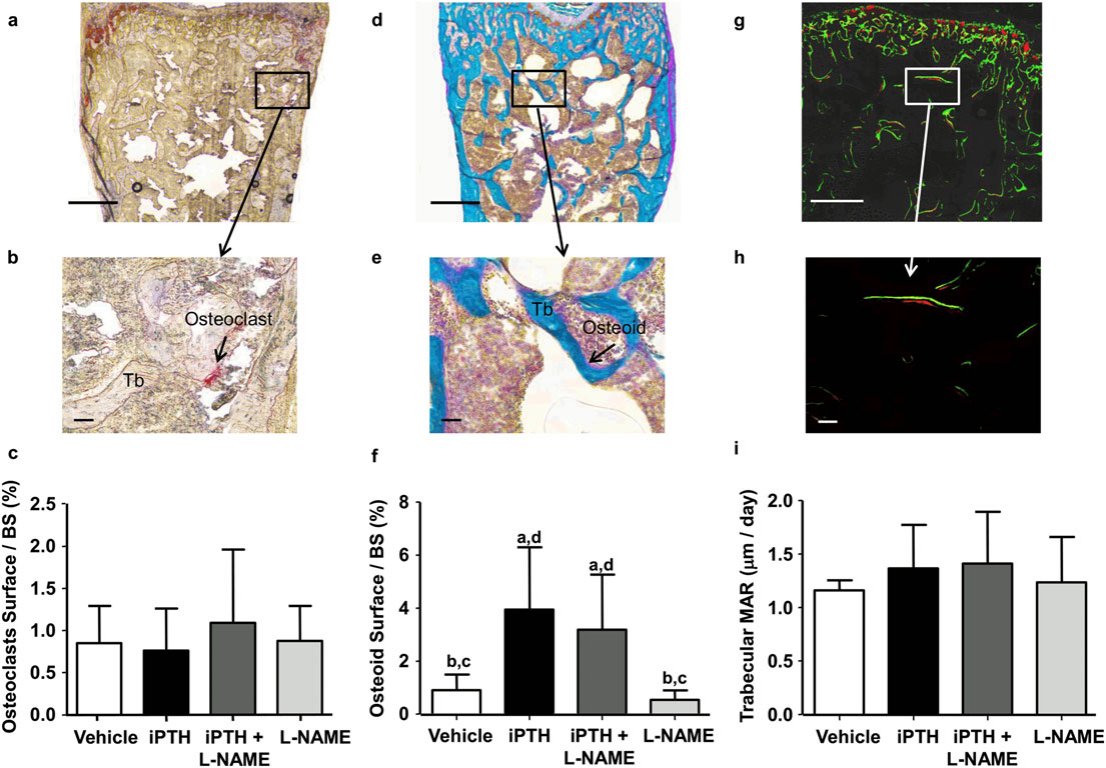
Effects of PTH and L‐NAME on trabecular bone turnover. (a) Representative brightfield micrographs of non‐decalcified sections of secondary spongiosa in the distal femur stained for TRAP (a) or Goldner's trichrome (d) with respective magnified regions demonstrating (b) TRAP‐positive osteoclasts (arrow) or (e) positively stained osteoid (arrow) lining trabeculae (Tb). (g) Representative confocal micrographs of a non‐decalcified section of the secondary spongiosa of the distal femur, showing the fluorescent labels (calcein, green and xylenol orange, red, 5 days between both labels) marking bone apposition during the last 5 days of the 28 day dosing experiment. Scale: bars = 500 µm in top row images and bars = 50 µm in second row images. Bar graphs (C, F, I) summarize the different histomorphometric parameters in trabecular bone at the end of the 28‐day treatment with vehicle (control), PTH 1–34 (80μg/kg/d) and L‐NAME (30 mg/kg/d), alone or in combination including: (c) percentage of osteoclast surface per bone surface, (f) percentage of osteoid surface per bone surface and (i) mineral apposition rate (MAR). Values are mean ± SD, *n* = 7–8 mice/group; ^a^ *P* < 0.05 versus control; ^b^ *P* < 0.05 versus iPTH; ^c^ *P* < 0.05 versus iPTH + L‐NAME, ^d^ *P* < 0.05 versus L‐NAME

In order to determine osteoblast and osteoclast activities, we also examined histomorphometric parameters of trabecular bone in the distal femur metaphysis. The percentage of resorption surfaces was not significantly different among groups (Figure 6 a–c). The proportion of osteoid surfaces was significantly greater in both iPTH (+167%) and PTH + L‐NAME treated groups (+133%) than control (Figure 6 d–f). Although L‐NAME co‐administration appeared to reduce the elevation of osteoid surfaces compared to iPTH alone, this did not reach statistical significance. L‐NAME treatment alone did not affect any of the cellular parameters. Despite the significant increase in osteoid surfaces induced by iPTH, the bone apposition rate (MAR) in trabecular bone was not affected by any of the treatments (Figure 6 g–i). Thus, iPTH appear to regulate bone remodelling by altering the thickness and number of trabeculae without inducing a final increase in trabecular bone volume fraction compared to control. Co‐administration of L‐NAME did not reduce iPTH‐induced trabecular thickening but did further decrease the number of trabeculae which resulted in a final reduction of the trabecular bone volume fraction.

### L‐NAME co‐administration does not modify iPTH‐enhanced bone mechanical strength

In order to explore whether these changes in perfusion and associated modifications in bone formation and geometry engender a structural advantage, we measured whole‐bone mechanical properties by three‐point bending. This showed that iPTH significantly increased several extrinsic bone material properties compared to control (Table 2), including yield load (+17%) and ultimate load (+15%), which measure whole femur strength. Surprisingly, this iPTH‐induced enhancement in bone strength was not reduced by co‐administration of L‐NAME. Moreover, iPTH and L‐NAME in combination exerted unexpected effects on some of the intrinsic material properties of bone, including Young's modulus, yield stress and ultimate stress, which were significantly increased compared with bones from both control and also with iPTH‐treated groups (Table 2). Conversely, iPTH did not affect any of the intrinsic material properties of cortical bone compared to control. Finally, L‐NAME alone did not affect either extrinsic or intrinsic material properties of bone compared with control. These data indicate that the combined effect of opposing modifications in structural versus material properties induced by iPTH versus those in the presence of PTH + L‐NAME caused the two treatment groups to achieve a similar improvement in femur mechanical strength compared with control.

**Table 2:** Mechanical properties of the femur measured by the three‐point bending test. Bone mechanical properties were measured in femurs dissected at the end of the 28‐day dosing for the four treatment groups (*n* = 7‐8 mice/group): Vehicle (control), iPTH, iPTH + L‐NAME and L‐NAME alone

**Group**	**Control**	**iPTH**	**iPTH + L‐NAME**	**L‐NAME**
Diameter femurs (mm)	1.16 ± 0.06	1.22 ± 0.04	1.18 ± 0.03	1.19 ± 0.05
Yield load (N)	12.0 ± 1.3^b^	14.1 ± 1.0^a^	14.6 ± 1.5^a^, ^c^	12.5 ± 1.3^b^
Ultimate load (N)	13.5 ± 1.0^b^	15.5 ± 0.7^a^	16.6 ± 1.1^a^, ^c^	14.9 ± 1.7^b^
Breaking load (N)	10 ± 2	12 ± 2	11 ± 2	10 ± 4
Yield extension (mm)	0.18 ± 0.02	0.19 ± 0.03	0.18 ± 0.01	0.17 ± 0.03
Ultimate extension (mm)	0.26 ± 0.05	0.25 ± 0.03	0.24 ± 0.03	0.24 ± 0.05
Breaking extension (mm)	0.5 ± 0.1	0.4 ± 0.2	0.5 ± 0.1	0.4 ± 0.1
Stiffness (N/mm)	70 ± 6	80 ± 7	87 ± 10^a^	79 ± 11
Elastic modulus (MPa)	4763 ± 945	4530 ± 654^b^	5580 ± 516	5037 ± 363
Yield stress (MPa)	69 ± 9^b^	72 ± 6	83 ± 10^a^, ^c^	71 ± 8^b^
Ultimate stress (MPa)	78 ± 10^b^	79 ± 5^b^	93 ± 6^a^	84 ± 8
Breaking stress (MPa)	58 ± 9	61 ± 13	60 ± 7	57 ± 22
Yield strain	0.015 ± 0.002	0.017 ± 0.003	0.016 ± 0.001	0.015 ± 0.002
Ultimate strain	0.022 ± 0.005	0.023 ± 0.002	0.021 ± 0.003	0.021 ± 0.003
Breaking strain	0.04 ± 0.01	0.04 ± 0.02	0.04 ± 0.01	0.04 ± 0.01
Work to fracture (mJ)	4.3 ± 1.3	4.3 ± 2.2	5.1 ± 0.7	4.0 ± 1.7
Work yield to fracture (mJ)	3.3 ± 1.3	3.0 ± 2.2	3.8 ± 0.7	3.0 ± 1.7

Values are mean ± SD;

a *P* < 0.05 versus control;

b *P* < 0.05 versus iPTH + L‐NAME;

c *P* < 0.05 versus L‐NAME.

## Discussion

The results of the present study show that PTH (1–34) causes acute stimulation of limb blood flow in BALB/c mice, and that this response is required for PTH to achieve its anabolic effect on cortical bone. PTH treatment however did not increase trabecular bone volume in BALB/c mice. Our data also shows that the vasomotor effect of PTH is not diminished by repeated daily dosing. This observation suggests that the transient surge in blood perfusion following daily PTH administration may be integral to the mode of action of intermittent PTH on bone.

We demonstrate that blockade of nitric oxide synthase activity by the inhibitor L‐NAME greatly reduced PTH‐stimulated blood flow and also restricted the anabolic effect of PTH on cortical bone. These results fit with the earlier finding that PTH‐induced acute vasodilation in rat femoral principal nutrient arteries *in vitro* was almost totally inhibited by L‐NAME 26, and suggest a significant role for NO‐mediated arterial vasorelaxation in the osteoanabolic actions of PTH. Bone cells also signal through NO 36, 37, and thus specific interactions between PTH and NOS in bone cells cannot be ruled out, although *in vitro* evidence suggests that NO production is not significantly altered by PTH in human and murine osteoblasts 38, 39. Our results showed that L‐NAME treatment alone did not significantly affect perfusion or bone mass, suggesting that NOS function may not play an important role in bone homeostasis in unchallenged adult mice. This is consistent with another report showing that L‐NAME administration for 18 days did not significantly change trabecular bone formation rate in rats 40.

Because our laser Doppler imaging measurements only incorporated perfusion near the surface of mouse hindlimbs (to a maximum penetration depth in skin/muscle of about 2 mm), we tested whether acute vasodilation also induced an increase in intraosseous cortical perfusion. The reference method to measure bone blood flow is the intravascular injection of labelled microspheres^41^. However, a lot of studies demonstrated a positive correlation between the standardized measure of blood flow with labelled microspheres and blood perfusion measured by the Laser Doppler Imaging^42^, demonstrating that the whole hindlimb blood flow is a good indicator of bone blood flow. We found that daily PTH treatment increased cortical bone perfusion (assessed by procion red staining), consistent with previous studies reporting a PTH‐induced elevation of radioactive microspheres uptake in the tibiae of rats 43. Our results are also consistent with the recent findings of Roche *et al.* 22, who showed that tibial perfusion and blood vessel area increased in response to daily PTH injection. In contrast, though, we did not observe any chronic changes in mouse hind limb perfusion, measured by laser Doppler, following prolonged daily administration of PTH. However, because of technical limitations, we were not able to measure cortical perfusion at the end of our study, and thus cannot exclude the possibility that iPTH might have a chronic effect on this parameter.

Our finding that the anabolic effect of iPTH on cortical bone was not completely blocked by L‐NAME suggests that other bone and/or vascular responses are triggered by the activation of PTHR1 receptor independently of NOS activity. One possibility is the involvement of VEGF signalling, because a VEGF‐blocking antibody has been reported to abolish the anabolic effect of PTH and impair bone blood vessel remodelling *in vivo* 23, as well as reducing vasodilation in *vitro* 26. Alternatively, and irrespectively of the changes in perfusion, PTH may act on osteocytes to decrease production of the Wnt signalling and bone formation inhibitor, sclerostin 7. Our observation that L‐NAME inhibition of the osteoanabolic effect (MAR and BFR/BS) of iPTH on cortical bone was more prominent on endosteal surfaces fits the view that the vascular effect of PTH is particularly marked in more hypoxic areas of bone such as the endosteum and less essential at more oxygen‐replete periosteal surfaces 35. This differential stimulation of bone formation in response to the different treatments on cortical bone external and internal envelops suggests a complex remodelling of the mineral surfaces of these envelops, which may contribute to changes in the material properties and, ultimately, the mechanical strength of bone.

Notwithstanding its inhibition of PTH‐induced cortical osteogenesis, L‐NAME co‐administration did not affect the mechanical strength of the femurs. The reasons for this are not completely clear but it could be that the reduction in PTH‐induced cortical osteogenesis by L‐NAME was compensated by other changes in the intrinsic mechanical properties of the bones in order to achieve the same overall mechanical strength 44. Indeed, we observed that L‐NAME treatment improved the intrinsic mechanical properties of the cortical bone compared to control, while PTH alone had no effect. However, we were unable to detect any changes in either BMD or cortical porosity by microCT in PTH and L‐NAME‐treated groups. Alternatively, it is possible that any changes in mechanical strength are below the powers of detection of this methodology.

We did not observe any effect of iPTH on the trabecular bone volume fraction (BV/TV) in the distal femoral metaphysis. We noted a similar lack of effect of iPTH in the trabecular bone of the L5 vertebra (data not shown), suggesting that this was not a site‐specific phenomenon. This lack of effect was surprising, given that several reports have documented a strong increase in trabecular bone mass in C57Bl/6 mice administered similar doses of iPTH for 15 to 28 days 22, 45, 46. It was however shown that the responsiveness to anabolic actions of PTH is mouse strains dependent, 47 and it is likely that our results may reflect responses that are specific for the BALB/c mouse strain, consistent with a previous report 48. It is also possible that our lack of effect of iPTH on trabecular BV/TV may be related to the age of the animals studied, because iPTH has been shown to increase metaphyseal trabecular bone in younger BALB/c mice 49. The lack of BV/TV elevation by iPTH in our study did not appear to reflect the complete absence of an effect but rather an homeostatic response where the increase in trabecular thickness (Tb.Th) was compensated by a decrease in the number of trabeculae (Tb.N). Moreover, the proportion of trabecular surfaces covered by osteoid was increased by iPTH and unaffected by co‐treatment with L‐NAME, suggesting that iPTH augmented trabecular bone matrix formation independently of NOS activity. This increase was not matched, however, by an accrual in mineralized bone matrix. Additionally, the reduction in Tb.N in the iPTH treated mice does not fit with the lack of change in osteoclast surfaces. The reasons for the lack of concordance of histomorphometric and microCT parameters in trabecular bone are unclear.

In conclusion, by demonstrating that the acute vasorelaxation induced by PTH modulates its anabolic effect on cortical bone in BALB/c mice, the current study provides new evidence for a novel mode of action for iPTH in bone, through an indirect, transient vasomotor stimulation which intermittently increases bone perfusion. Our simplest explanation of this action is that the daily boost of oxygen and nutrients induced by iPTH in bone might be responsible for accelerating bone cell metabolism and, ultimately, increasing bone formation. In addition, changes in intraosseous perfusion are bound to alter gradients for hypoxia in the bone marrow with the potential of activating quiescent osteoblasts and/or mobilizing osteogenic progenitor cells 50. Our results support the notion that daily pharmacological stimulation of vasodilation could either boost the effect of osteoanabolic drugs or by itself increase bone formation and bone mass.

## Conflict of Interest

None of the authors have a conflict of interest.
